# Amino Acids 785, 787 of the Na^+^/H^+^ Exchanger Cytoplasmic Tail Modulate Protein Activity and Tail Conformation

**DOI:** 10.3390/ijms222111349

**Published:** 2021-10-21

**Authors:** Xiuju Li, Tommy Tu, Sicheng Quan, Francisco J. Quintero, Richard Fahlman, Larry Fliegel

**Affiliations:** 1 Department of Biochemistry, University Alberta, Edmonton, AB T6G 2H7, Canada; xjli@ualberta.ca (X.L.); tu1@ualberta.ca (T.T.); quan@ualberta.ca (S.Q.); rfahlman@ualberta.ca (R.F.); 2 Instituto de Bioquímica Vegetal y Fotosíntesis Consejo Superior de Investigaciones Científicas, Avda, Américo Vespucio 49, 41092 Sevilla, Spain; fjquintero@ibvf.csic.es

**Keywords:** Na^+^/H^+^ exchanger, pH regulation, phosphorylation, intracellular pH, membrane protein

## Abstract

The mammalian Na^+^/H^+^ exchanger isoform 1 (NHE1) is a plasma membrane protein ubiquitously present in humans. It regulates intracellular pH by removing an intracellular proton in exchange for an extracellular sodium. It consists of a 500 amino acid membrane domain plus a 315 amino acid, regulatory cytosolic tail. Here, we investigated the effect of mutation of two amino acids of the regulatory tail, Ser^785^ and Ser^787^, that were similar in location and context to two amino acids of the *Arabidopsis* Na^+^/H^+^ exchanger SOS1. Mutation of these two amino acids to either Ala or phosphomimetic Glu did not affect surface targeting but led to a slight reduction in the level of protein expressed. The activity of the NHE1 protein was reduced in the phosphomimetic mutations and the effect was due to a decrease in Vmax activity. The Ser to Glu mutations also caused a change in the apparent molecular weight of both the full-length protein and of the cytosolic tail of NHE1. A conformational change in this region was indicated by differential trypsin sensitivity. We also found that a peptide containing amino acids 783–790 bound to several more proximal regions of the NHE1 tail in in vitro protein interaction experiments. The results are the first characterization of these two amino acids and show that they have significant effects on enzyme kinetics and the structure of the NHE1 protein.

## 1. Introduction

The mammalian Na^+^/H^+^ exchanger (NHE1) is a ubiquitously expressed membrane protein of human cells. It removes a single intracellular proton in exchange for one extracellular sodium ion. NHE maintains intracellular pH (pH*_i_*), protecting cells from acidification resulting from metabolism. It also responds to osmotic challenge regulating cell volume [1,2]. There are nine SLC9A type isoforms of NHE, two SLC9B types, and also two SLC9C types. NHE1 is the primary plasma membrane isoform found in all mammalian cells while most other isoforms have restricted cellular locations or intracellular locations [2,3,4,5,6,7,8]. NHE1 is the ubiquitous plasma membrane isoform in mammals with several important physiological functions [3,4,5,9,10,11,12]. NHE1 consists of two domains, a membrane transport domain that moves ions and a cytosolic regulatory domain. The N-terminal membrane domain is 500 amino acids and its structure has recently been determined [13]. The cytosolic regulatory domain is 315 additional amino acids and functions to regulate the membrane domain [14]. NHE1 is critical in heart disease in both heart hypertrophy, and ischemia-reperfusion damage [15]. Elevation of NHE1 activity accentuates the deleterious role the protein plays in some forms of heart disease [16,17]. NHE1 is also important in breast cancer, where it acts as a trigger for metastasis [18,19,20]. Here again, elevated activity of the protein promotes the disease, and phosphorylation at a specific amino acid in the tail is suggested to be a critical trigger enhancing NHE1 activity and promoting the disease [21,22]. Thus, it seems clear that regulation of the NHE1 at its cytosolic tail is important in its function and human disease. At the same time, while direct inhibition of NHE1 has been suggested to treat human disease, there have been detrimental off-target side effects of NHE1 inhibitors in at least one clinical trial [23] and modification of NHE1 through its regulation has been suggested to be one approach for the treatment of human disease [24].

Regulation of NHE1 by phosphorylation has, in particular, been shown to affect NHE1 function. Phosphorylation was earlier suggested to account for about half of the growth factor-induced regulation of NHE1 and was thought to occur in the distal 180 amino acid region of the carboxyl-terminal tail [14,25]. Briefly, some of the well-characterized protein kinases that are implicated in regulation through phosphorylation of the cytosolic domain are β-Raf (Thr^653^) [26], p38 MAPK (Thr^718^, Ser^723^, Ser^726^, Ser^729^, rabbit sequence changed to equivalent human) [27], and protein kinase B/Akt (Ser^648^), [28,29]. Erk-mediated regulation of NHE1 has also been shown to act through Ser^703^ [30] and several other amino acids are also implicated including Ser^693^, Ser^723^, Ser^726^, Ser^770^, Ser^771^, Thr^779^, and Ser^785^ [31,32,33,34]. Phosphorylation was first shown to cause structural changes in the cytosolic regulatory domain in 2013 [35]. More recently, the C-terminus has been suggested to contain in part, intrinsically disordered regions that are changed to more ordered regions upon ERK-dependent phosphorylation [36]. 

Amino acid Ser^785^ has been shown to be phosphorylated by ERK2 [36] and contains an “SP” consensus site. Interestingly, it is followed by Ser^787^. These two amino acids are similar to a phosphorylation site in the Na^+^/H^+^ exchanger SOS1 of *Arabidopsis*, which is important in the regulatory activity of this protein by its own protein kinase SOS2 (unpublished observations) [37]). Preliminary experiments in our laboratories have identified the equivalent residues, Ser^1136^ and Ser^1138^, of SOS1 as being phosphorylated by MPK6 and the SOS1 kinase, SOS2. In this study, we examined the effect of phosphorylation of the equivalent two residues of NHE1—Ser^785^ and Ser^787^. We examine the effect of mutation of these two amino acids on activity, expression, targeting, structure, and intramolecular interactions of the tail domain. These results show that these two amino acids are important in the structure and activity of the protein and cause a change in the conformation of the C-terminal regulatory region. 

## 2. Results

**Figure 1:**
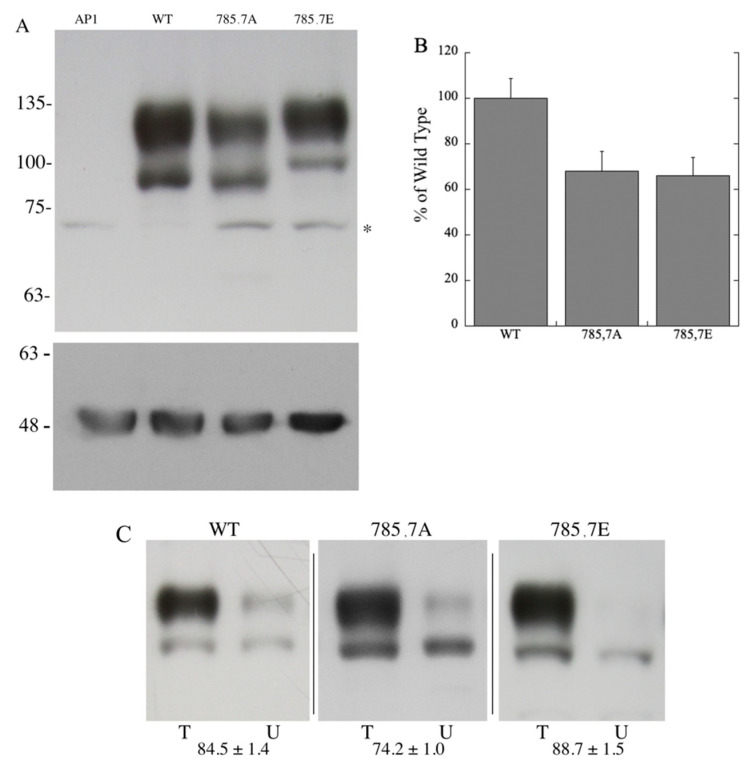
Expression and targeting of wild-type NHE1 and mutant NHE1 proteins. (**A**) Upper panel, Western blot of whole-cell lysates of stably transfected AP1 cells containing the wild-type (WT) or indicated NHE1 proteins. Asterisk indicates non-specific immunoreactive band present in all cell lysates. WT, wild type. AP1, mock transfected AP1 cells. Lower panel, blot re-probed with anti-tubulin antibodies. (**B**) Bar graph illustrating relative expression levels of WT, 785A, 787A mutant cells and 785E, 797E containing cells. Results are the mean ± S.E. of at least four experiments. (**C**) Cell surface localization of wild-type and mutant Na^+^/H^+^ exchanger proteins expressed in AP-1 cells. Equal amounts of total cell lysate (T, left lane) and unbound intracellular lysate (U, right lane) were examined by Western blotting with anti-HA antibody. The percent of the total NHE1 protein found on the plasma membrane is indicated for each cell type and are the mean ± S.E. n ≥ 3 determinations. Samples for each type of protein are from different Western blots (indicated by dividing line).

To investigate the role of amino acids 785 and 787 in the function and activity of the protein, we examined the effect of mutagenesis of these two amino acids to either Ala or to the phosphomimetic mutation Glu. Stable cell lines were made of the mutants to allow for more precise analysis of the mutations, similar to previously published work [38]. We initially examined expression levels, and surface targeting of the wild-type and mutant NHE1 proteins expressed in AP-1 cells (Figure 1). Expression levels were determined by Western blotting using an antibody directed against the HA tag present on the C-terminus of the Na^+^/H^+^ exchanger protein. Figure 1A demonstrates that NHE1 is expressed as a higher molecular weight form that is fully glycosylated and a lower possibly de-glycosylated form, similar to results described earlier [38]. The mutant protein with amino acids Ser^785^ and Ser^787^ changed to Glu, was noticeably different from the wild-type protein, with a larger apparent molecular weight. This reduction in mobility was not apparent with mutation of the amino acids to Ala. The expression level of both mutants was slightly, but not significantly, reduced in comparison to the wild-type protein. 

The targeting of NHE1 was analyzed by biotinylating cell surface proteins and analyzing cell surface localization as described earlier [38]. Wild-type NHE1 protein targeted about 80% to the cell surface (Figure 1B). Minor variations were found between the WT and mutant proteins, but these were not significantly different than the wild-type NHE1 protein.

**Figure 2:**
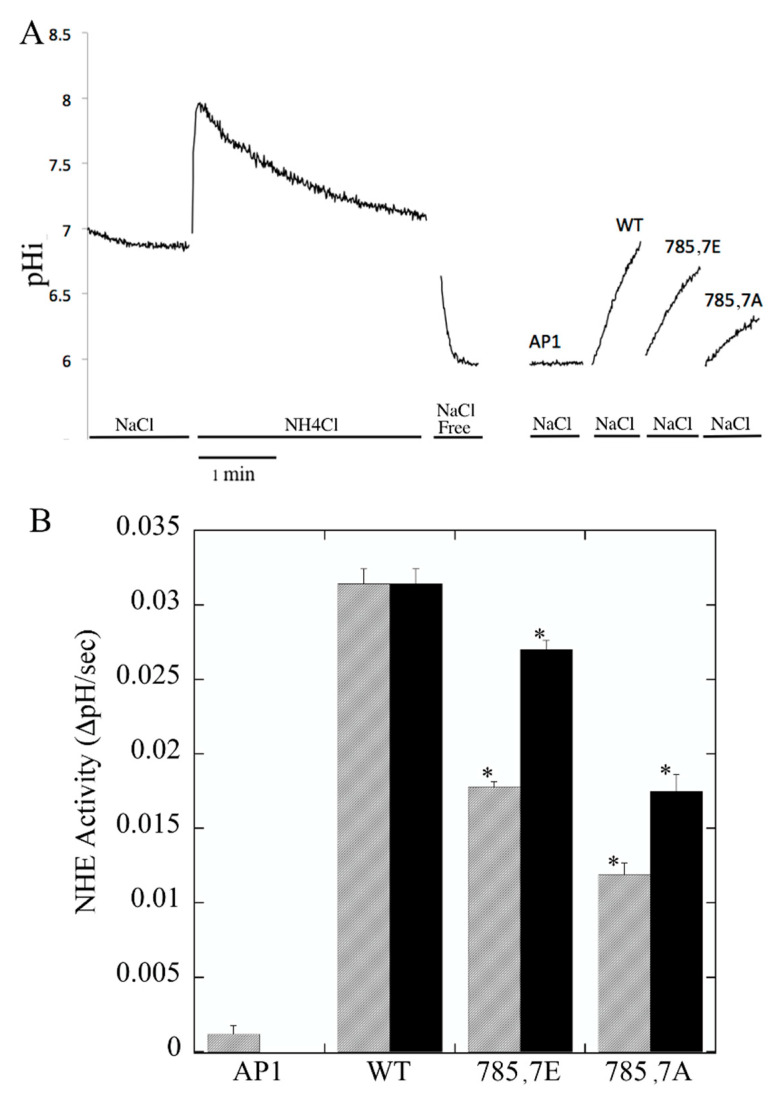
Na^+^/H^+^ exchanger activity of AP1 cells stably transfected with wild-type (WT) and mutant NHE1 proteins. Na^+^/H^+^ exchanger activity was measured after ammonium chloride induced acid load as described in Section 4. (**A**) Examples of measurement of Na^+^/H^+^ exchanger activity. A Trace is shown for one ammonium chloride treatment of cells and the recovery only is shown for one of each kind of cell type. AP1 are cells without the NHE1 protein. (**B**) Summary of activity of NHE1 expressing stable cells lines and AP1 cells. All results are the means ± S.E. of at least six determinations. Results are shown for mean activity of both uncorrected (hatched) and normalized for surface processing and expression levels (solid bars). Asterisks indicate that the value is significantly different than appropriate matched wild-type NHE1 protein at *p* < 0.001.

We next examined the activity of the Na^+^/H^+^ exchanger in the wild-type and mutant-containing cell lines, with and without corrections for levels of expression and surface targeting. Figure 2A shows an example of the activity. After cells equilibrate from BCECF loading, NH_4_Cl is added which causes an initial alkalinization. After three minutes, NH_4_Cl equilibrates inside the cell and the media was changed to sodium-free media to induce acidosis. The addition of NaCl allows recovery from the low pHi. The wild-type protein showed a robust recovery from intracellular acidosis with NaCl addition. AP1 cells showed little recovery from acidosis—less than 5% of the recovery rate of cells containing the wild-type protein. Cells containing the mutant proteins showed reduced activity. Figure 2B shows a summary of the uncorrected results and the results corrected for the changes in the level of expression and targeting of the mutant proteins. Both mutants had a reduction in activity compared to controls, and this persisted even when corrected for the levels of protein and targeting.

**Figure 3:**
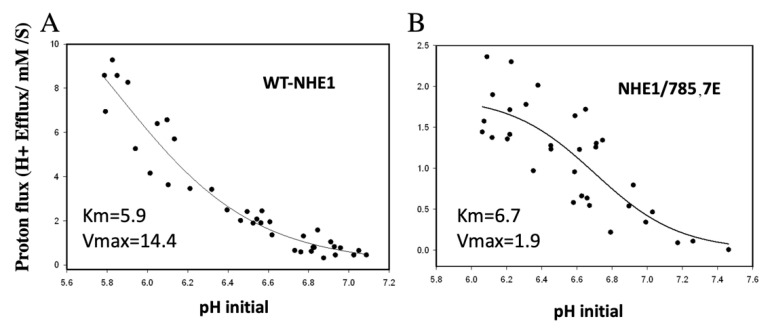
Dependence of proton efflux on intracellular pH in NHE1 expressing AP1 cells. Cells were incubated with varying amounts of ammonium chloride that was removed to induce different degrees of acid load. Recovery was determined in Na^+^-containing buffer 135 mM. Proton efflux was calculated from the initial rate of recovery and the buffering capacity as described in Section 4. WT, wild type, mutants 785E, 787E where indicated. (**A**) Proton flux wild type NHE1 containing cells and (**B**) proton flux of 785E, 787E NHE1-mutant containing cells.

To examine the activity of NHE1 in protein in more detail, wild-type and mutant 785E, 787E NHE1-containing cells were acidified to varying degrees as described in the Section 4. Proton flux was then calculated using the buffering capacity of the particular cell type. The results are shown in Figure 3. Wild-type NHE1 had a Vmax of proton flux of approximately 10, while the mutant was approximately 1/5 of that at about 1.8. In contrast, the kinetics of activation of the pH sensitivity of the mutant were different from those of the wild type. The Km was shifted to a more alkaline pH, making the protein more active at more alkaline pHs. 

**Figure 4:**
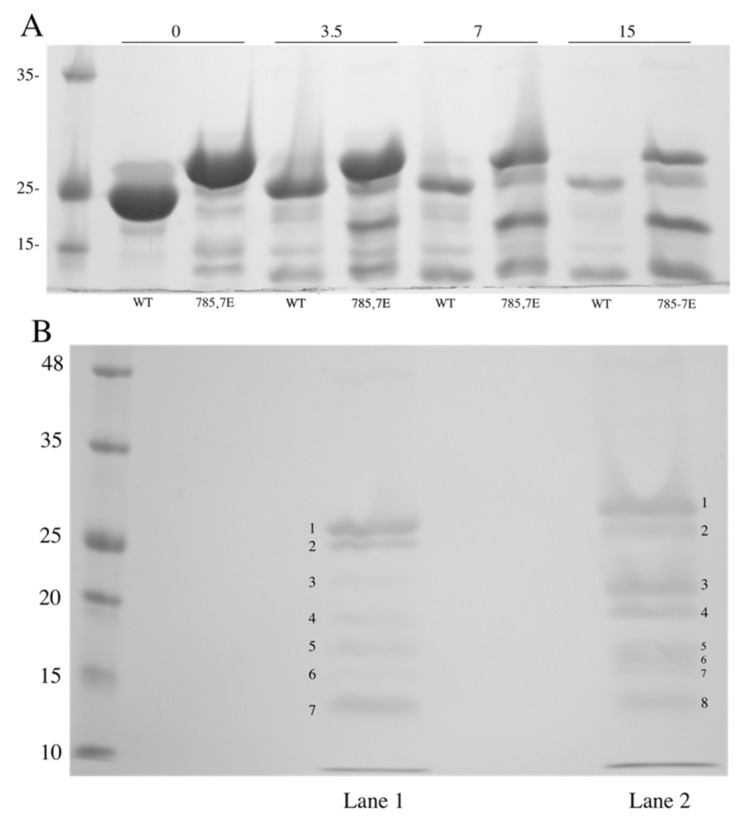
Differential mobility and trypsin sensitivity of wild-type (WT) and mutant NHE1 proteins. (**A**) His-182 NHE1 protein and His-182 with the 785E, 787E mutations were purified as described in Section 4 and treated with increasing amounts of trypsin (3.5, 7, and 15 ng) for 30 °C × 5 min. Reactions were terminated with SDS-PAGE sample buffer and run on 12% PAGE. (**B**) Tryptic digest of 35 μg of wild-type (Lane 1) or phosphomimetic (Lane 2) His-182 protein with 10 ng trypsin for 10 min (30 °C). Fragments excised for mass spectrometry are indicated.

To further characterize the effect of mutations to amino acids 785 and 787 on the NHE1 carboxyl-terminal region, we expressed and purified the C-terminal 182 amino acids of the protein with and without the phosphomimetic mutations. We then treated the proteins with varying amounts of trypsin and examined the time course of degradation and protein fragments produced. The results are shown in Figure 4A. Without added trypsin, the wild-type protein ran as a protein band with a molecular weight of approximately 25 kDa. In contrast, the mutant protein had reduced mobility and was approximately 29 kDa in size. The pattern of proteins produced with trypsin treatment varied between the two proteins. Most notable was a protein with an apparent molecular weight of approximately 19 kDa that was produced in the mutant protein and not in the wild type. 

**Figure 5:**
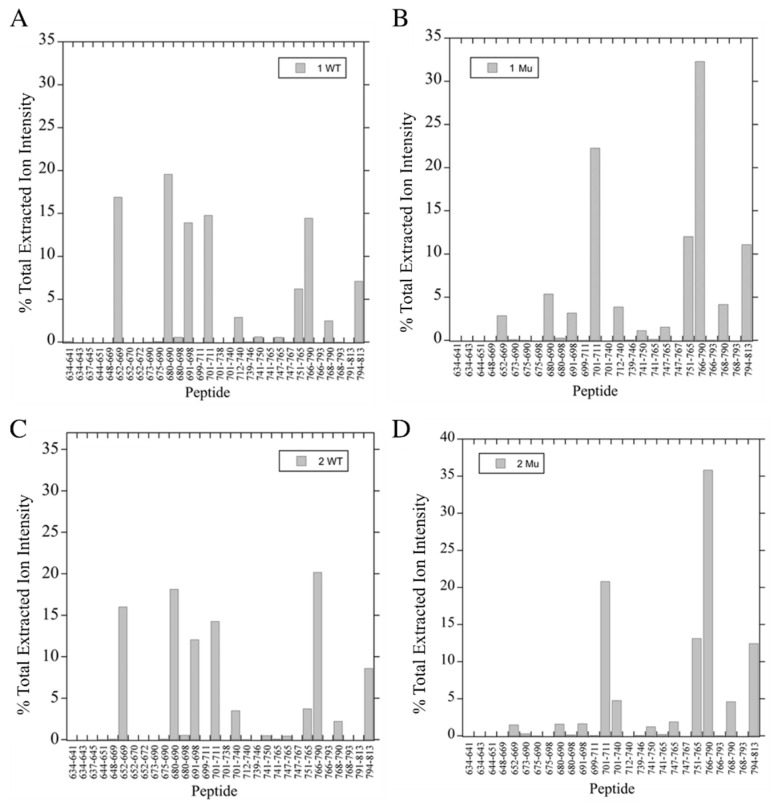
(**A**–**D**) Mass spectrometry analysis of tryptic fragments of bands 1 (**A**,**B**) and 2 (**C**,**D**) of wild-type (**A**,**C**) and phosphomimetic mutant (**B**,**D**) His-182 proteins. The bands were excised and treated with trypsin and fragments were analyzed by mass spectrometry as described in Section 4. The sum of the total extracted ion intensity was used to plot the observed relative amount of each fragment. WT, wild-type His-182 protein, Mu, phosphomimetic mutant His-182 protein. The theoretical peptides are shown on the X-axis. % of total fragments found is plotted for each peptide.

Further tryptic analysis of the wild-type and phosphomimetic NHE1 was followed by mass spectrometry of the in-gel fragments that were treated with trypsin. We then attempted to identify the fragments produced by further limited trypsin treatment. Figure 4B shows an SDS-PAGE analysis of trypsin treated wild-type (lane 1) and mutant phosphomimetic (lane 2) His182 protein. Numbering indicates the fragments excised from the gels and subjected to trypsin treatment followed by mass spectrometry to identify fragments produced. For the full-length His 182 wild-type and mutant proteins and the next largest fragment of each, mass spectrometry analysis is shown in Figure 5A–D. The major tryptic fragments of the wild-type protein were of amino acids 652–669, 680–690, 691–698, 701–711, and 766–790. Here, these all comprised between 12 and 20% of the total fragments produced (Figure 5A,C). The digestion pattern of the phosphomimetic His182 was different (Figure 5B,D). Here, the peptide 766–790 was released in greater relative amounts, comprising over 30% of the released peptides. Additionally, the 701–711 and 751–765 peptides were a higher relative amount of peptide in both the phosphomimetic full-length His182 protein and the next smallest protein. The results of the fragmentation analysis of the remaining protein bands of Figure 4B are shown in Supplementary Figure S1. It should be noted that bands 5 and 7 of the wild type, appeared to be a mixture of more than one protein. Similarly, bands 5–8 of the mutant were not well distinguished from one another so there was no further analysis of the patterns of the fragments obtained with further digestion.

**Figure 6:**
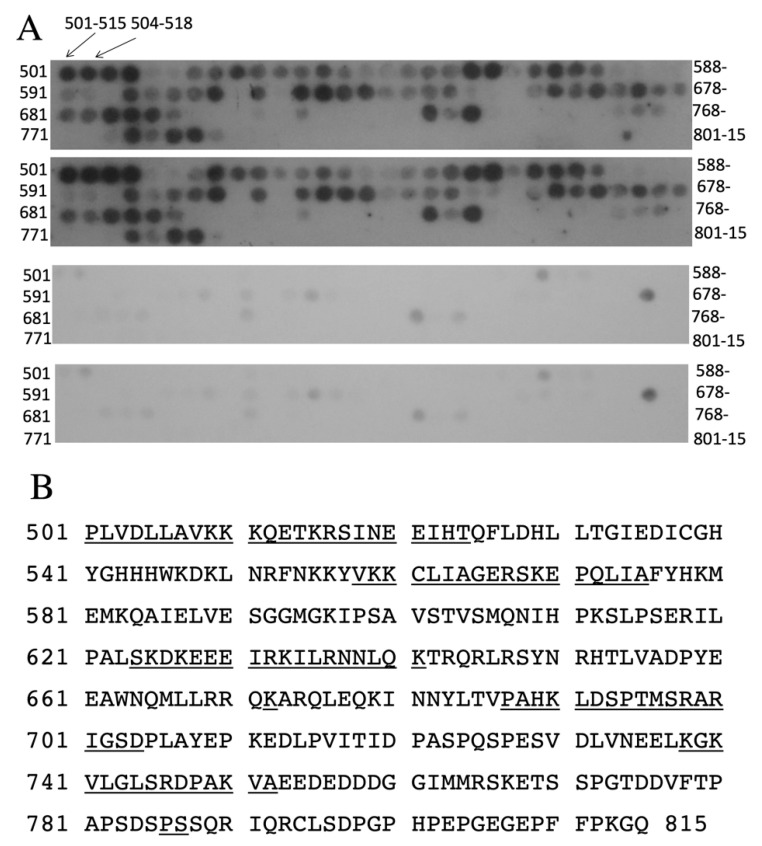
Probing of peptide constructs [39] of amino acids 501–815 of the human NHE1 C-terminus with the peptide of the sequence N-GSD**E**P**E**SQGDYKDDDDKG or with the control peptide of the sequence N-GSD**A**P**A**SQGDYKDDDDKG. Underlined sequences indicate the amino acids of the 15mer that bound to the probe that bound in larger amounts. Bold amino acids indicate either phosphomimetic replacements (Glu) or Ala for amino acids Ser^785^ and Ser^787^. The N-terminal peptide of the peptide array is indicated. Each spot on the array contains a peptide of 15 amino acids in length. The adjacent peptide is shifted by three amino acids as indicated. (**A**) Illustration of results of probing with peptides (in duplicate). Upper two panels are separate IQ-peptide arrays probed with experimental phosphomimetic peptide. Lower two panels are separate IQ-peptide arrays probed with the control peptide which had two Glu residues changed to Ala. (**B**) NHE1 C-terminal amino acids indicating regions of stronger binding to the tagged peptide.

To examine if the amino acids of the region 783–790 could bind to other parts of the NHE1 carboxyl-terminal tail, we made a synthetic peptide of this region containing the phosphomimetic amino acids. We then used it to probe an oligopeptide array of amino acids 501–815 of the human NHE1 C-terminus. The synthetic peptide had amino acid equivalents Ser^785^ and Ser^787^ changed to Glu, to act as phosphomimetics. The results are shown in Figure 6. There were several discrete regions where the 783–790 peptide bound to the peptide array—these are summarized in Figure 6B. Regions of stronger, more apparent binding were mainly between amino acids 500 and 630 and tended to be positively charged. A control peptide, with amino acid equivalents Ser^785^ and Ser^787^ mutated to Ala, was used to probe the lower two oligopeptide arrays. This oligopeptide bound to the peptide array in much lower amounts. There were very few regions of binding to the array, and these were in much lower amounts than with the phosphomimetic peptide. 

## 3. Discussion

Regulation of NHE1 by phosphorylation affects NHE1 function. While some amino acids of the regulatory cytosolic region have been shown to be phosphorylated, the full extent of phosphorylation and the means by which phosphorylation affects NHE1 function is not yet clear. Here, we examined amino acids Ser^785^ and Ser^787^ of the regulatory cytoplasmic domain of NHE1. Initially, we demonstrated that mutation of these residues had functional effects on the activity of the protein. Mutation to either Ala or the phosphomimetic mutation to Glu decreased the activity of the protein. In the case of the mutation to Glu, the result was surprising. Kinetic analysis of the protein showed that this was mostly due to a decrease in the Vmax of the protein. However, there was also a shift to a more alkaline affinity, which in fact indicated more activity at more alkaline intracellular pHs. 

The complex effect noted here, led us to examine in more detail how the putative phosphorylation-mediated effects could be occurring. We noted that even in the full-length NHE1 protein, changing Ser*^785^* and Ser*^787^* to Glu created a significant mobility shift. The change was much greater than any change made in the mass of the protein (Figure 1A) suggesting a conformational change that affected mobility, that was stable enough to be detected in SDS-PAGE. To analyze this in more detail, we made large amounts of the C-terminal 182 amino acids of NHE1, with or without the change in amino acids Ser^785^ and Ser^787^ to Glu. Here again, there was a noticeable change in the mobility of the protein (Figure 4) suggesting a conformational change. To examine this in more detail and to characterize the conformational changes, we carried out a time course of limited trypsin digestion of the C-terminal wild-type and phosphomimetic protein and found different production rates of proteolytic products from the proteins. It was notable that a tryptic peptide of amino acids 766–790, containing amino acids Ser^785^ and Ser^787^ mutated to Glu, was more rapidly released compared to the same peptide in the wild-type protein. This peptide made up a greater relative amount of the released peptides in the mutant compared to the wild type (Figure 5A–D, Supplementary Figure S1). This suggests that the region had become more accessible to trypsin in the phosphomimetic, relative to the wild-type protein indicating a change in conformation relative to the wild type. 

As the cytosolic tail is known to regulate the activity of NHE1, it was also of interest to determine if amino acids of this region could bind to other parts of the NHE1. Figure 6 demonstrates that a synthetic peptide containing the phosphomimetic mutations binds to several groups of amino acids. Notably, this includes a proximal region of amino acids 501–524. Therefore, it is possible that the region containing amino acids, when phosphorylated, binds to this proximal region and affects NHE1 activity. Amino acids 501–524 are predicted to be close to the lipid bilayer [40] which might indicate they are in proximity of the intracellular face of the pore of the protein. The binding of a more distal region of the cytoplasmic tail could certainly affect transport, and in this study, we showed that these mutations do modulate NHE1 function. How the cytosolic tail modulates the membrane domain at the molecular level is not known. It has been shown that for potassium channels, the tail can inactivate the channel and can also modify channel activity. Phosphorylation-dependent regulation of the channel’s activity also occurs [41]. Future studies will examine the mechanism by which the tail regulates the membrane domain. 

Ser*^785^* has been shown to be phosphorylated by ERK2 [31,36]. The alkaline shift in activity caused by mutation of this residue would be consistent with stimulation of NHE1 by ERK2 [42]. The reason for the reduction in Vmax activity is not yet known but could be due to some kind of incomplete activation of the protein. ERK phosphorylates NHE1 in multiple locations [31,36] and here we only examined the effect of phosphomimetic mutations on one localized region of the protein, rather than multiple sites. Nevertheless, our results show that this region of the protein is capable of affecting transport and exhibits a change in conformation. Additionally, we demonstrate that the phosphomimetic protein can bind to other regions of the NHE1 cytosolic domain. We suggest that these amino acids are part of a regulatory phosphorylation cascade with other amino acids. When phosphorylated, a local change in conformation is induced. This causes this region to interact with proximal membrane amino acids and affects NHE1 activity. We note that phosphorylation of amino acids Ser^770^ and Ser^771^ has also been shown to induce conformation changes in the cytosolic tail [35]. Together with Ser^785^ and Ser^787^, such changes may mediate hormonal regulation of NHE1. Amino acids Ser^785^ and Ser^787^ fall within a flexible disordered region of the cytosolic tail of NHE1 [2]. One possibility is that phosphorylation at these sites induces structure in this region. It is interesting to note that the phosphatase calcineurin has been shown to dephosphorylate the nearby amino acid, Thr^779^ though it did not dephosphorylate Ser^785^ with comparable avidity. Dephosphorylation of amino acid Ser^787^ was not tested in this study. Ser^787^ was near a TxxP motif, (^779^TPAP^782^) which is a substrate for calcineurin [34]. Future studies should examine if calcineurin can also dephosphorylate this nearby residue. 

## 4. Materials and Methods

### 4.1. Materials

Synthetic oligonucleotides for site-specific mutagenesis were obtained from IDT (Coraiveille, IA, USA). Sulfo-NHS-SS-biotin was purchased from Thermo Fisher (Waltham, MA, USA). 2′,7-bis(2-carboxyethyl)-5(6) carboxyfluorescein acetoxymethyl ester (BCECF-AM) was purchased from Molecular Probes, Inc. (Eugene, OR, USA). Lipofectamine^TM^ 2000 reagent was purchased from Invitrogen Life Technologies (Carlsbad, CA, USA). Sigma-Aldrich (St. Louis, MI, USA) or Fisher Scientific, (Toronto, ON, Canada) supplied other chemicals of analytical grade. The plasmid pYN4+ has a hemagglutinin (HA) tag, is described earlier [43], and was used to express cDNA for the full-length human NHE1 protein. The Ni-NTA resin was obtained from QIAGEN (Toronto, ON, Canada). 

### 4.2. Cell Culture and Stable Transfection

A cell line deficient in its endogenous NHE1, AP1 cells, was used to characterize the activity of the wild-type protein vs. mutant Na^+^/H^+^ exchangers. AP1 cells were made from Chinese hamster ovarian cells [44]. AP1 cells were stably transfected with LIPOFECTAMINE™ 2000 reagent [44]. The pYN4+ plasmid contains a neomycin resistance cassette. This allows the selection of transfected cells with G418 antibiotic. Cell lines were re-established from previously frozen stocks at between passage numbers 5–11. For each mutant, at least two stable cell lines were independently made [44]. 

### 4.3. Plasmid Mutations

The plasmid pYN4+ contains the full-length human cDNA of NHE1 with a C-terminal HA tag. Site-directed mutagenesis was used as described earlier [45]. The primer CCCGCGCCCAGTGACgcgCCggcCTCCCAGAGGATACAGCGC and its complement were used to mutate amino acids Ser^785^ and Ser^787^ to Ala residues. A silent NaeI site (underlined) was introduced to aid in screening. Another primer CCCGCGCCCAGTGACgatCCggaCTCCCAGAGGATACAGCGC and its complement were used to change Ser^785^ and Ser^787^ to Asp residues. A new BspE1 site was used introduced in this case. 

### 4.4. Cell Surface Expression

We used sulfo-NHS-SS-biotin to label the cell surface proteins in order to examine targeting of the NHE1 protein [46]. Total cell proteins were then solubilized and cell surface proteins including NHE1 were removed using immobilized streptavidin resin. We immunoblotted against immunoreactive HA-tagged NHE1 protein thereby examining removal of cell surface proteins with streptavidin-agarose [47,48]. The levels of the immunoreactive protein were quantitated using ImageJ 1.35 software (National Institutes of Health, Bethesda, MD, USA). Quantification was of both the upper and lower HA-immunoreactive bands of the NHE1 protein. The percentage of the protein targeting to the cell membrane was calculated using this equation: ((Total-unbound)/Total) × 100%.

### 4.5. SDS-PAGE and Immunoblotting

We examined NHE1 levels in stably transformed AP1 cell lines using immunoblotting against the HA tag on the NHE1 protein [49]. Cell lysate proteins from the cell lines were run on SDS-PAGE (10%) gels and were transferred to nitrocellulose membranes. One hundred micrograms of protein of control or experimental lysates were run in triplicate for quantification. The BioRad D/C^TM^ Protein Assay Kit was used to measure protein concentrations. The secondary antibody was a peroxidase-conjugated goat anti-mouse antibody or anti-rabbit antibody (Bio-Can, Mississauga, ON, Canada). Protein quantification was performed as above. 

### 4.6. Intracellular pH Measurement

Cells were grown to approximately 80–90% confluence on glass coverslips in order to measure the pH_i_ and NHE1 activity. BCECF was loaded into cells as described earlier [50] and intracellular pH fluorescence was measured using a PTI Deltascan spectrofluorometer. An acute acid load was induced with ammonium chloride to measure NHE1 activity [51]. Ammonium chloride (50 mM × 3 min) addition was followed by removal to induce acute acidosis. Change in the pH_i_/s during the first 20 s of recovery in NaCl-containing medium was measured. An internal pH_i_ vs. fluorescence calibration curve was performed for every sample by using nigericin [44]. Results were the mean ± S.E. of at least eight experiments. Statistical significance was determined using the Wilcoxon–Mann–Whitney test. 

To measure proton flux and affinity of wild-type and mutant NHE proteins, initially the buffering capacity of the cells was determined as described earlier [50,52]. Cells were then acidified to varying degrees using different amounts of ammonium chloride and the activity of the protein was measured. Proton affinity and maximum flux were calculated as described earlier [50,52,53]. 

### 4.7. Expression and Purification of the NHE1 C-Terminal Regulatory Region

Na^+^/H^+^ exchanger fusion proteins. The carboxyl-terminal 182 amino acids of the human Na^+^/H^+^ exchanger (NHE1) was expressed as a fusion protein with a C-terminal histidine tag (His-182) using the plasmid pDest 14 and the GatewayTM Cloning System as described earlier [54,55]. The same cDNA was made again using a mutant form of the Na^+^/H^+^ exchanger using the 785E, 787E as a template for PCR. Protein purification was via immobilized metal (nickel) affinity chromatography essentially as described earlier [56]. For trypsin digestion samples were incubated at 30 °C for 5 min with the indicated amount of trypsin. The reaction was terminated by the addition of SDS-PAGE sample buffer and run on 12% SDS-PAGE. 

### 4.8. Peptide Blot

A series of IQ-peptide constructs of amino acids 501–815 the human NHE1 C-terminus were made similar to earlier [39]. The peptides of 15 amino acids in length were made and the next one made was shifted by three amino acids along the NHE1 sequence. They were synthesized on cellulose membranes that were acid-hardened and derivatized with a polyethylene glycol spacer. To probe the peptide array, a synthetic peptide was made of the sequence N-GSD**E**P**E**SQGDYKDDDDKG. Underlined residues encoded for the amino acids 783–790 of NHE1 with phosphomimetic Glu residues replacing Ser^785^ and Ser^787^. The C-terminal 9 amino acids coded for a Flag tag which was used for detection. To probe the array, the array was activated with methanol for 1 min, then washed twice with water. Then, it was equilibrated with binding buffer (50 mM Tris-HCl pH 7.5, 150 mM NaCl, 0.1% Triton X 100) for 2 hr at room temperature. This was followed by blocking for 1 hr in binding buffer with 1 μM BSA. The array was then incubated at 4 °C with binding buffer with the synthetic peptide (100 μM) overnight. It was washed three times with binding buffer, 10 min each, and then incubated with antibody against the Flag tag. Immunodetection was with enhanced chemiluminescence as described earlier [42]. A control peptide N-GSD**A**P**A**SQGDYKDDDDKG was used to probe IQ blots and to examine the effects of neutralization of the charge of the phosphomimetic Glu residues, which were changed to Ala residues. 

### 4.9. Mass Spectrometry

The indicated protein bands were digested with trypsin in-gel. Briefly, the excised bands were de-stained twice in 100 mM ammonium bicarbonate/acetonitrile (50:50). The samples were then reduced with 10 mM βME in 100 mM ammonium bicarbonate and alkylated with 55 mM iodoacetamide in 100 mm bicarbonate. After dehydration, the gel segments were digested with trypsin (6 ng/μL) overnight at room temperature. The tryptic peptides were then extracted from the gel with a 97% water/2% acetonitrile/1% formic acid solution. This was then followed by a second extraction using 50% of the first extraction buffer and 50% acetonitrile. 

The fractions containing tryptic peptides were then dried under vacuum and were dissolved in 5% *v*/*v* acetonitrile and 1% *v*/*v* formic acid and were resolved and ionized by using nanoflow HPLC (Easy-nLC II, Thermo Scientific, Waltham, MA, USA) coupled to the LTQ XL-Orbitrap hybrid mass spectrometer (Thermo Scientific). Nanoflow chromatography and electrospray ionization were carried out using a PicoFrit fused silica capillary column (ProteoPepII, C18) which had a 100 μm inner diameter (300 Å, 5 μm, New Objective). The peptide mixtures were injected onto the column using a flow rate of 3000 nL/min and were then resolved at 500 nL/min using 70 min linear gradients from 0 to 45% *v*/*v* aqueous ACN in 0.2% *v*/*v* formic acid. The mass spectrometer was operated in a data-dependent acquisition mode, which recorded high-accuracy and high-resolution survey Orbitrap spectra using external mass calibration. The resolution was 60,000 and used an *m*/*z* range of 400 to 2000. The fourteen most intense multiply charged ions were then sequentially fragmented using collision-induced dissociation. The spectra of their fragments were recorded in the linear ion trap. After two fragmentations, all precursors selected for dissociation were dynamically excluded for 60 s 

The data were processed using Proteome Discoverer 1.4 (Thermo Scientific) and SEQUEST was then used to search the Uniprot Human Protein Database (Thermo Scientific). The search parameters included a fragment mass tolerance of 0.8 Da and a precursor mass tolerance of 10 ppm. Peptides were then searched using the dynamic modifications of carbamidomethyl cysteine as a static modification and oxidized methionine and deamidated glutamine and asparagine. 

## Data Availability

Not applicable.

## Supplementary Materials

All data are available online at https://www.mdpi.com/article/10.3390/ijms222111349/s1.
