# Supplementary figures and images for: Amino Acids 785, 787 of the Na+/H+ Exchanger Cytoplasmic Tail Modulate Protein Activity and Tail Conformation
Source: Int J Mol Sci. 2021 Oct 21;22(21):11349. doi: 10.3390/ijms222111349 (PMC8583816; doi:10.3390/ijms222111349)

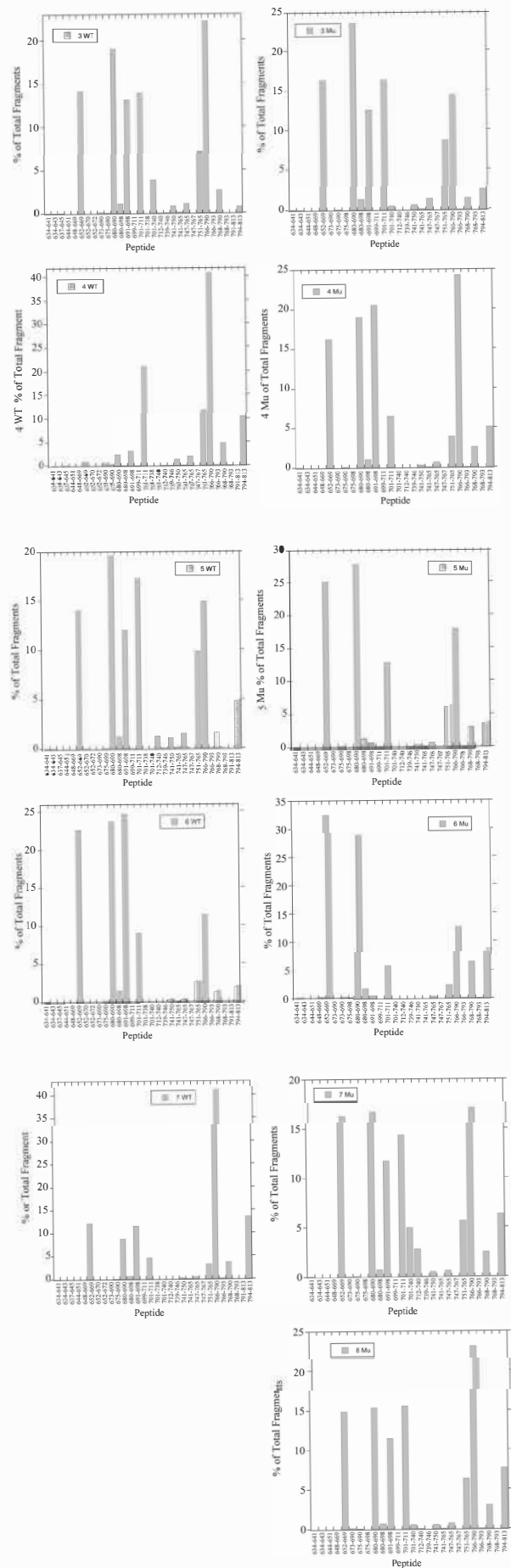

Figure S1. Fragmentation analysis of protein bands.

Supplement: Supplementary file 1 [file ijms-22-11349-s001.zip › ijms-1409190-supplementary.pdf]
